# Firm’s compliance behaviour towards food fortification regulations: Evidence from oil and salt producers in Bangladesh^☆^

**DOI:** 10.1016/j.foodpol.2021.102143

**Published:** 2021-10

**Authors:** Amrita Saha, Daniele Guariso, Mduduzi N.N. Mbuya, Ayako Ebata

**Affiliations:** a Institute of Development Studies, University of Sussex. BN19RE, United Kingdom; b Department of Economics, Jubilee Building, University of Sussex, BN19SL, United Kingdom; c The Global Alliance for Improved Nutrition (GAIN), 1701 Rhode Island Ave NW, Washington, DC 20036, United States

**Keywords:** Micronutrient, Firms, Premix, Nutrition, Bangladesh

## Abstract

Impact of national food fortification programs is contingent on the extent to which there is compliance with national standards. However, this compliance is often sub-optimal and is not consistently measured. One of the challenges to more regular measurement is an over-reliance on quantitative assessments of micronutrient levels for compliance, which are costly. In resource constrained environments, this contributes to weaknesses in regulatory monitoring. We offer an alternative, systems-based approach to determine compliance, presenting a unique score that can capture firms’ compliance behaviour, based on whether and how firms actually carry out stages of the fortification process. The key utility of such a measure being its use to monitor fortification propensity and assess changes in response to interventions. Further, we present an empirical application of this measure, providing novel evidence on firms’ compliance towards food fortification regulations in Bangladesh, investigating the institutional and firm-level factors that correlate with compliance behaviour towards food fortification regulations among edible oil and salt producers.

## Highlights

• An alternative, systems-based approach to determine compliance with food fortification, based on the fortification process.
• More aware and engaged firms have a better understanding of the policy that helps recognise the value of complying.
• Greater frequency of face-to-face interactions is linked to better compliance for recently introduced regulations.
• Firms’ distrust in the effectiveness of incentives and penalties can hamper compliance.
• A sustainable longer-term method could combine frequent monitoring of compliance behaviour with occasional product testing.

## 1 Introduction

Large-scale fortification of staple foods and condiments is a cost-effective, scalable and evidence-based strategy to help meet human requirements for essential nutrients and address micronutrient deficiencies, when delivered as intended (Horton, 2006, Keats et al., 2019). An important factor determining the impact of national food fortification programs is the extent to which there is compliance with national standards. However, this compliance is often sub-optimal, limiting potential for impact; and is often not consistently measured, also limiting the ability of program managers to implement course corrective actions. One reason for this is an over-reliance on quantitative tests of nutrients as the definitive indicator of compliance because they offer numerical results that can be compared (directly or indirectly) with the micronutrient specifications. Specifically, verifying legal compliance involves product sampling across different batches at the production site to produce composite samples that are tested for nutrient content in accredited laboratories (Rimkus, 2021). While important, over-reliance on such an approach is prohibitive in terms of costs and availability of resources, with resulting weaknesses in regulatory and performance monitoring (Luthringer et al., 2015, Rowe et al., 2018).^1^

Current policy guidance (GAIN and PHC, 2018) recommends a different paradigm, in the form of a systems-based approach to determine compliance, emphasizing the process of fortification over regular testing of fortified food samples; including specific components, such as a comprehensive audit checklist that covers food quality, food safety and use of the premix to determine whether the fortification process is sufficiently adding micronutrients to foods. However, to the best of our knowledge, there is no existing methodology to operationalize this recommendation or devise an alternative indicator of compliance.

To address this gap in the literature, we present a new methodological approach that can capture firms’ compliance behaviour to food fortification, based on whether and how firms actually carry out the stages of any fortification process. We draw from Henson and Heasman (1998), and frame firm-level decision to comply with regulations for food items as a multi-stage process, where in the first stage, the firm engages with the regulation, and, in the second stage, the firm decides whether to fortify food items, based on perceived costs and profits of compliance and on incentives and penalties, and, if so, how. In capturing compliance behaviour, we specifically focus on the steps following a firm’s decision to comply which constitute the different stages of a standard compliance process, the implementation and monitoring related to the fortification, targeting the technologies and processes adopted by the firm. The score itself comprises general components for fortification related with the premix; storage; different equipment, its use, maintenance and calibration; and internal monitoring. Further components can be added to these general ones, depending on the product and specific nature of the fortificant.

The utility of such a measure is threefold. First, it can be used by fortification program stakeholders to monitor fortification and assess changes in response to interventions such as capacity building. Second, it can provide a proxy for assessing compliance, providing a less costly, human resource and technical capacity intensive alternative to compliance measurement. Third, it can serve an explanatory purpose by assessing the components of compliance related behaviours that predict compliance or non-compliance in a given context and hence inform targeted corrective actions.

Furthermore, building on such a compliance measure, a sustainable longer-term compliance method could combine frequent monitoring of the compliance behaviour score with occasional quantitative product testing. For product sampling, products have to be analysed in accredited labs, following strict protocols. With further testing and validation, our approach can reduce the frequency of such sampling/testing such that the legally enforceable inspections occur with lower frequency. We would argue that this would be a positive, so that the inspections can be more preventive and cooperative than punitive and enforcement focused. Such a protocol would be relatively more cost-effective,^2^ and would also be likely to yield greater and more accurate data on compliance from firms. Testing this combination is however outside the scope of this study but remains a future direction for further research.

Although many low- and middle-income countries (LMICs) have adopted food fortification as a vehicle to improve nutrition, firms’ level of compliance with fortification standards varies greatly across country contexts (Aaron et al., 2017, Luthringer et al., 2015). We apply our methodology to oil and salt fortification in Bangladesh, where fortification of salt with iodine and edible oil with Vitamin A has been mandatory since 1989 and 2013, respectively (Aaron et al., 2017, Knowles et al., 2017). However, in 2015, only 65% of households had salt with some added iodine, whereas 51% had salt that was adequately iodized (Knowles et al., 2017). Similarly, a market assessment conducted in 2017 revealed that 59% of oil available in Bangladesh is fortified, (Jungjohann et al., 2019, Jungjohann et al., 2021). Further, there is a stark difference between packaged and bulk oil in terms of fortification status: while 95% of packaged oil was found to be appropriately fortified, only 41% of bulk oil met the standard (GAIN and icddr’b, 2017). These findings indicate suboptimal compliance and a missed opportunity to tackle high prevalence of micronutrient deficiency in Bangladesh: 20.6% and 40.0% of children of school age were Vitamin A and iodine deficient, respectively, according to the Bangladesh Micronutrient Survey of 2011–2012.

Our study makes a twofold contribution. First, we provide a general methodological innovation to accurately measure compliance with processes needed to meet food fortification standards without the need for costly micronutrient quantification; Second, we present novel evidence applying this method for Bangladesh, and draw attention to key institutional and firm-level factors for firms’ compliance towards food fortification regulations among edible oil and salt producers. The paper is structured as follows. Section 2 provides an overview of the literature on fortification and motivates the framework; Section 3 outlines the conceptual basis for the new compliance score; Section 4 provides details of the research methodology, including the survey design, variables and key hypotheses; Section 5 presents the results including the descriptive statistics on key variables and analysis of the factors that correlate with firms’ compliance behaviour; Section 6 discusses the results in terms of the wider context of the study and key policy implications; and Section 7 provides concluding remarks.

## 2 Food fortification – Framework

Food fortification law is an example of regulation that aims to improve public goods – in this case the nutritional outcomes of the population. Existing research highlights the necessary conditions for private firms– whose main objective is to generate profit – to comply with regulations aimed at increasing public goods and specifically nutritious food items. These include effective enforcement of regulations, the existence as well as adequate distribution of economic benefits gained from complying with regulations, and the technical capacity to comply (Henson and Heasman, 1998, Maestre et al., 2017). In addition, effective enforcement of regulations creates incentives for firms to comply as failing to do so can lead to significant economic loss for violating firms because of penalties and recalls, alongside disclosure of food scandals, which results in negative publicity and loss of consumer trust (Henson and Hooker, 2001, Hollweg, 2019). Evidence indicates that there are two levels of factors influencing firms’ compliance with fortification laws: *firm-level factors and institutional factors*. Below, we elaborate on these two levels of factors that incentivise for-profit firms to comply with fortification regulations.

### 2.1 Firm-level factors

**Fig. 1:**
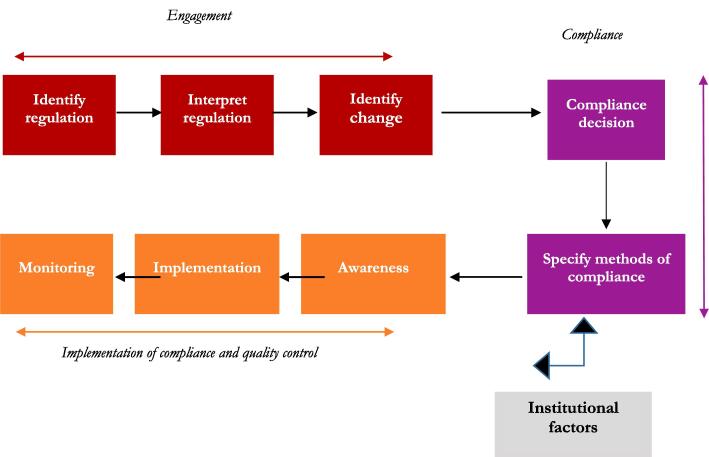
Compliance process for firms.

Source: Adapted from Henson and Heasman (1998).

Henson and Heasman (1998) suggest that firm-level decision to comply with regulations for food items is a multi-stage process (Fig. 1).

The first stage for the firm is *engagement* with the regulation - identifying the regulation, understanding the details, and identifying what changes need to be made to the current operation practices. Then, the firm decides whether or not to comply, and if so how. Subsequently, the proposed changes in operation need to be communicated internally and the compliance decision is taken. Then, the implementation is done and finally monitored (*implementation of compliance and quality control*). Motives, attitude and capability of the firm have been demonstrated to be important influences on the likelihood of compliance or non-compliance (Kagan and Scholz, 1980).

Small- and medium-scale producers may be disadvantaged in acquiring information about and engaging with regulations (i.e. the first three steps in the above process) (Yapp and Fairman, 2006). This is because small-scale firms may lack staff capacity to constantly monitor changes in the regulatory environment (Henson and Heasman, 1998, Yapp and Fairman, 2006). Additionally, firms may struggle to identify relevant sources of information regarding regulation when the roles and responsibilities of regulation development and enforcement may not be well communicated (Luthringer et al., 2015).^3^ Drawing on this, the key hypothesis of the paper examines the relationship between engagement, as outlined in the framework above, with food fortification and firm compliance behaviour.

Hypothesis 1 (H1) Firms’ engagement with food fortification, which includes knowledge around the issues surrounding fortification and its regulation, as well as the health implications and current consumption levels of fortified food are important factors explaining firms’ compliance.

Once firms gather needed information about the regulations, in the second stage they decide whether to fortify food items, and, if so, how. Firms make decisions based on the perceived costs and profits of compliance (Henson and Heasman, 1998). In terms of the cost of fortification, high cost of premix has been identified as a key constraint (Luthringer et al., 2015). This issue is particularly challenging in LMICs, as premix is sold by multi-national corporations (Food Fortification Initiative, 2019) and has to be imported to these countries (Garrett et al., 2016), firms – and in particular small-scale ones (Nyumuah et al., 2012) – face challenges in acquiring premix at a low price (SPRING, 2018). This may incentivise using sub-standard, and therefore cheaper, premix and/or applying less than the recommended quantity.

In markets where a fortification law has only recently been introduced, producers of fortified products often face competition from non-fortified counterparts (Abdoulaye and Manus, 2018, Luthringer et al., 2015). In these new markets for fortified food items, awareness among consumers regarding the benefits of micronutrients may be low (Ara et al., 2010) and labelling of fortified products is unclear (Whiting and Calvo, 2006). As a result, producers may find that there is insufficient market demand for fortified products, and therefore are not incentivised to comply with the fortification law. In addition, profit margin is thin because many actors exist in a market (USAID and GAIN, 2015). As a result, firms are disincentivised to invest in premix and equipment needed for fortification.

The literature on fortification patterns in LMICs suggests that firms struggle to comply with standards even when they decide to fortify (Osendarp et al., 2018; Maestre and Poole, 2018). For example, based on a study of salt iodization in Pakistan, Masuood and Janjua (2013) found that salt mills lacked processing equipment that ensured uniform mixing of iodine in salt. In Nigeria, firms faced difficulty in accessing high-quality, and therefore expensive, premix and adequate technology to mix fortificant in food items (Ogunmoyela et al., 2013). Likewise, a study on wheat millers in Pakistan found that, although millers own fortification equipment, it was of sub-optimal quality and at times was installed incorrectly (Randall and Anjum, 2014).^4^ Oil refineries, on the other hand, did not add fortificants consistently, leading to varying levels of fortification (Randall and Anjum, 2014). In addition, storage condition are sometimes inappropriate, which could lead to deterioration of fortificant before reaching consumers (Darnton-Hill, 1998).

### 2.2 Institutional factors

The second set of factors influencing firms’ compliance with fortification laws is regulatory institutions and their influence on firm behaviour (Fig. 1). Regulation enforcement shapes firm behaviour by setting rules and instituting mechanisms (i.e. incentives and penalties) to ensure that they comply with these rules (Graham and Woods, 2006, Rowe et al., 2018). Indeed, inspection positively influences firms’ compliance with regulations that enhance public goods (Gupta et al., 2019). However, many government agencies face challenges in effectively enforcing rules. One key factor is a lack of human and financial resources, which hampers the frequency of inspection (Garrett and Luthringer, 2015). Shortage of staff means that regulatory authorities may find it difficult to monitor often very numerous small-scale firms across a country (Chadare et al., 2019). In addition, a high turnover of staff implies that the skills and know-how of fortification monitoring are quickly lost (WHO, 2006). This results in infrequent and ineffective visits at production sites by regulators. Therefore, a second hypothesis of this study examines how frequency of inspections from the regulatory authority influences firms’ compliance.

Hypothesis 2 (H2) Frequency of inspections by the regulatory authority is linked positively with firms’ compliance.

Even when regulators detect under-compliance or non-compliance, there may be a lack of consequences to this behaviour, such as when fines imposed on producers are too low (Luthringer et al., 2015). Sometimes, regulators keep fines low due to concerns about the political cost of harsh regulation (Luthringer et al., 2015). This undermines compliance (Iscenko et al., 2016) as firms choose to pay the fine instead of upgrading their fortification equipment in order to avoid fines. Combined with infrequent inspections by regulators, firms do not perceive inspections as threatening. The third hypothesis of the paper interrogates the relationship between firms' perceived effectiveness^5^ of incentives and penalties and compliance behaviour.

Hypothesis 3 (H3) Firm’s perceived effectiveness of the penalties and incentives set by the government is linked positively with compliance.

Weak enforcement can be made more effective if regulators disclose firms’ non-compliance, leading to negative publicity and therefore a damaged reputation among consumers (Hollweg, 2019, Loader and Hobbs, 1999).^6^ However, for such pressure from consumers to materialise, the public must be aware of the fortification law in the first place, as well as the benefits of fortification. As this is often not the case (Ara et al., 2010), firms’ engagement with regulations, which includes knowledge around the issues surrounding fortification and its regulation as well as the health implications and current consumption levels of fortified food also remains low, which undermines their willingness to comply with the fortification law. In relation to this, we study the role of engagement for firms in mediating distrust of incentives and penalties.

Hypothesis 4 (H4) Firms’ food fortification engagement mediates distrust of incentives and penalties.

Overall, these dynamics crucially influence firm’s perception of costs and benefits of compliance (Henson and Heasman, 1998), and thereby their decision to comply.

## 3 Measuring compliance behaviour

In the context of food fortification, compliance is broadly defined as conformity to the micronutrient specifications detailed in the nationally adopted standards and to other food quality, safety, packaging, and labelling requirements (GAIN and PHC, 2018). These standards are often published as targets or actionable limits in the regulation or law, and the rules for enforcement are articulated. Additionally, the nodal agency for regulatory enforcement and monitoring is empowered to administer fines and penalties for non-compliance. In order to be deemed compliant, all food production facilities within the scope of the fortification regulations must ensure that their products conform to the micronutrient specifications detailed in the nationally adopted standards and to other food quality, safety, packaging, and labelling requirements. Determining whether a food manufacturing facility and its products are compliant is based upon the monitoring activities conducted by regulatory authorities. Before the actionable limits are published, government regulators need to agree upon and document the actions that will take place when quantitative results indicate that samples collected by inspectors from food production sites fall outside of the actionable limits. However, quantitative tests based on samples are often prohibitive, leading to severe weaknesses in regulatory monitoring (Luthringer et al., 2015, Rowe et al., 2018).

**Fig. 2:**
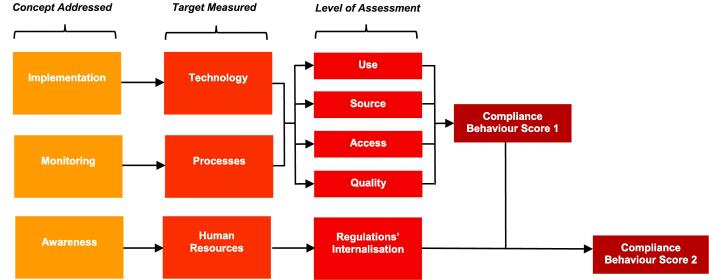
Compliance Behaviour Score Methodology.

A major challenge is measuring compliance behaviour that deviates from the regulation norms (i.e. non-compliance), as firms might be reluctant to self-report such behaviour. Firms are expected to report high compliance levels when asked about formal regulations or the implementation of these (IDS et al., 2020). In order to overcome this hurdle, we present a unique ‘*Compliance Behaviour Score’ (CBS)* that measures whether and how firms actually carry out stages of the fortification process in accordance with the framework outlined by Henson and Heasman (1998). Fig. 2 illustrates the compliance behaviour score methodology building on the stage of ‘implementation of compliance and quality control’: first, it outlines the three specific concepts (**Concept Addressed**) that are covered – *implementation, monitoring and awareness*; second, the target measurement that corresponds to each (**Target Measured**): technology for implementation, processes for monitoring, and human resources for awareness; finally, it presents the levels (**Levels of Assessment**) across the targets that may include use, access, source, quality and regulations' internalisation.

We create two different versions of the score. *CBS 1* is the main score that captures the different stages of a standard compliance process following a firm’s decision to comply. It captures the implementation and monitoring related to the fortification, targeting the technologies and processes adopted by the firm. The score comprises the following common components for fortificants (the full list included in this research is described in Appendix A): 1. Storage facility for premix; 2. Premix measurement equipment; 3. Mixers; 4. Fortification related IT use; 5. Testing samples (a. Single ‘grab sample’; b. Composite sample); 6. Recording of premix, mixing, final products, etc. (a. Premix stocktake; b. Premix calculations; c. Equipment maintenance; d. Equipment calibration; e. Mixing procedure; f. Frequency of sampling; g. Point of sampling; h. Test methods validation; i. Corrective actions).

For this research, applying the general methodology to edible oil and salt, the score for oil firms includes: 1. Titration equipment; 2. Blenders; while the score for salt firms includes: 1. Spraying technology; 2. Salt test kits; and 3. Salt iodization plants. Each of these technologies and processes is assessed at four levels: its use, access, source, and quality; giving us a comprehensive picture of the firm’s likely behaviour towards compliance. Combining the data from the various technologies and processes gave us the score for each firm. In our framework, the degree of compliance with mandatory fortification is captured by this compliance score.

We also create *CBS 2*, an alternate score that helps assess the robustness of the measurement. We consider this alternative definition that incorporates awareness of fortification regulations, targeting the firm’s human resources. This is assessed by how much the knowledge of the regulations is spread within the firm. Throughout the paper we will use CBS 1 as the main variable of interest, whereas CBS 2 will be employed to test the robustness of our baseline results.

Constructing a score that captures firm behaviour in relation to compliance is new in the literature on compliance with food fortification regulations. Given the novelty of the methodology, we perform various checks to examine the validity of this measure and its different definitions in our model. Through the above methodology we aim to provide a flexible framework that can be applied to different institutional contexts and industries. However, while we can assess the internal validity of this measure, examining its full external validity is a direction for future research, including further validation against measured micronutrient content to ascertain the full range of applications.

## 4 Data and methodology

### 4.1 Quantitative data

#### 4.1.1 Survey

A survey of edible oil and salt producers in Bangladesh was conducted from August to September 2019. This firm-level survey was designed to capture the main correlates of compliance (and non-compliance) with food fortification regulations, considering the specificities of these two industries in the Bangladeshi context.

The sampling frame consisted of 223 salt producers and about 80 oil producers (oil refineries and packers) in Bangladesh (IDS et al., 2020). The population of oil firms can be categorised into refineries (32%), registered packers (48%), and unregistered packers (20%). The target sample size for the survey was determined based on the following criteria: power calculations for drawing a statistically relevant number of firms that are representative of the edible oil and salt producers in Bangladesh; and expected response rates of firms and budget considerations.^7^

We began by conducting a pilot survey with 4 firms to understand the Bangladeshi context and inform the survey design based on findings from this exercise. Then, we targeted a random sample of 200 firms (68% of the total population), 150 salt firms and 50 oil firms (refineries^8^ and registered packers),^9^ drawn from the lists of firms prepared by the survey team as described earlier. Out of this sample, our data collection team completed a total 137 interviews with oil and salt firms (Table 1 below): 102 interviews with salt firms and 35 interviews with oil firms – approximately 70% of the total target population.^10^

**Table 1:** . Sample size – oil and salt.

Group	Total	Oil	Salt
Sample size (Achieved)	137	35	102
Target sample (% of population)	68%	70%	67%
Response rate (% of target)	70%	70%	68%

A key component of the survey was designed to measure food fortification engagement among firms, an element that stands out as crucial in the following analyses. Aside from standard characteristics – the type and level of production, human capital, and main reference markets – the survey was structured to gauge the most prominent factors associated with firms’ behaviour from the firm and institutional perspective. Firm-level factors include the characteristics of the premix supply, quality assurance standards, and human resources involved in the fortification process. Institutional features include the quality and characteristics of the normative environment (the frequency of inspections by the regulatory authority, sources of information on fortification requirements, the effectiveness of the incentives and penalties set by the government), but also the presence of external support.

**Fig. A1:**
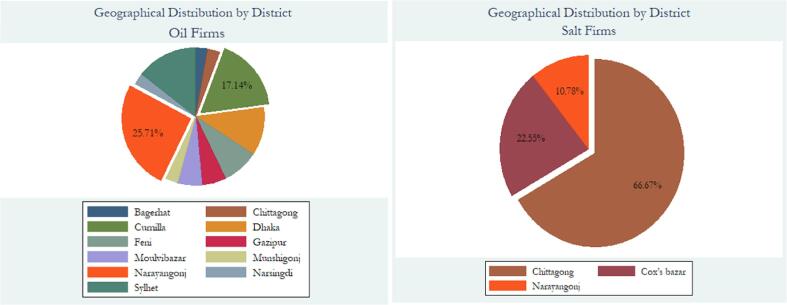
Geographical distribution of firms in study sample.

The geographical distribution of the sampled firms across various districts of Bangladesh (Fig. A1 in Appendix B) reveals that the salt firms are concentrated in Chittagong/Chattogram, Narayangonj, and Cox’s Bazar, while the oil firms are spread across different districts, but are mainly located in Narayangonj and Cumilla.

#### 4.1.2 Variables

A complete list of variables constructed and used for our analysis is outlined in Table 2 and discussed in detail below. We begin by outlining the dependent variables and the main variables of interest.

**Table 2:** . List of variables.

Variable	Description
**Dependent variables**	
**COMPLIANCE – MAIN**	
*Compliance Behaviour Score 1 (CBS 1)*	Score based on the use, access, source, and quality of the processes and equipment for fortification. Discrete variable running in the interval **[0,1]**.
*Comply*	Dummy variable – value of 1 if the *CBS 1* is higher than the average for oil and salt respectively, 0 otherwise**. [0 or 1]**.

**COMPLIANCE – OTHER**	
*Compliance Behaviour Score 2 (CBS 2)*	Score based on *CBS 1*, plus additional information on the human resources involved in the fortification process or aware of the fortification requirements. Discrete variable running in the interval **[0,1]**.

Independent variables [primary variables of interest]	
*Engagement*	Food fortification engagement measured as the number of correct answers to the questions on engagement and awareness. Discrete variable running in the interval **[0,5]**.
*Inspections*	The number of inspections by government authorities that firms tend to receive per year. Discrete variable.
*Inspection dummy*	Dummy variable – value of 1 if the number of inspections by government authorities is higher than the average for oil and salt, respectively. **[0 or 1]**.
*Effectiveness*	Average perceived effectiveness of the incentives and penalties set by the government. Discrete variable running in the interval **[0,5]**.
*Distrust*	Dummy variable – value of 1 if the average perceived effectiveness of the incentives and penalties set by the government is less than 4.5 (salt sample only). **[0 or 1]**.

Control variables	
*Employees*	Number of workers employed by the firm in 2018.
*Age*	Number of years since the establishment started operations.
*AvgInst*	Firm’s reported average degree of importance (on a scale from 1-Most important to 9-Least important) across three out of nine institutional factors that require improvement to ensure compliance with fortification standards (i.e., clear regulations, regulatory agency structure, and capacity)0.^1^**[1,9]**.

1 The full list of factors include: clear regulations; regulatory agency structure; regulatory agency capacity; regulatory agency financing; laboratory capacity; sampling/testing procedures; food industry engagement; enforcement (incentives/penalties); and communication between sectors.

##### 4.1.2.1 Dependent variables

*Compliance Behaviour Score 1 (CBS 1):* Compliance is identified using the tool designed to capture firm behaviour to comply with food fortification regulations in the Bangladesh context. For each technology/process relevant for fortification (as discussed in Section 3), we asked the following four questions for the four different levels of assessment:

• Use: Do you employ any of these? (1-Yes; 0-No)
• Source: Is it an in-house process or equipment? (1-Yes; 0-No)
• Access: Do you have access to it? (1-Yes; 0-No)
• Quality: How well do you think it is working? (Likert Scale of 1(Very bad)-5 (Very well))

**Fig. A2:**
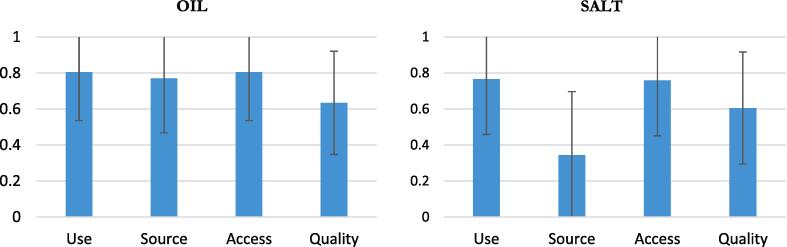
Intermediate scores underlying compliance score. Note: The bars report the mean and error bars report standard deviations.

We generate dummy variables for the first three questions and a normalized variable in the range 0–1 for the fourth question. We take the average across all technologies/processes to give us an intermediate score for each question (Fig. A2, Appendix B). The final score is the average of all the intermediate scores for each firm in the interval [0,1].

*Comply*: To construct a dummy variable that identifies compliance, we assume that values of the score higher than the average identifies firms that comply effectively with the fortification regulations, whereas being below the average means poor norm implementation. We create a dummy variable that takes the value of 1 if the CBS 1 is higher than the average for oil (0.75) and salt (0.62) respectively, or 0 otherwise.

*Compliance Behaviour Score 2 (CBS 2):* As discussed in Section 3, to check the validity of our main score, we also construct a second compliance score that in addition to the first one includes information on the human resources involved in the fortification process or aware of the fortification requirements. We asked the following two questions to capture regulations’ internalisation in relation to compliance:

• Does your firm have a designated compliance person / role? (1-Yes; 0-No)
• Who in your company needs to be aware of the mandatory fortification requirements? 1-Compliance staff; 2. Operational and plant managers; 3. Production staff; 4. Sales staff

We generate dummy variables for compliance person/role in the first question and dummy variables for categories of staff that are aware of the fortification requirements (1-Aware; 0-Not aware). We take the average across all categories of staff to create an intermediate score. The human resources score is the average of the first dummy variable and the intermediate score. The final score (CBS 2) is the average of CBS 1 and the human resources score.

##### 4.1.2.2 Independent variables

Next, we discuss the independent variables and the control variables.

*Engagement:* Food fortification engagement was captured by a distinct section of the survey that was intended to assess firms’ knowledge around the issues surrounding food fortification and its regulation. The following five questions were asked:

• Can you remember when mandatory fortification for salt/oil was first introduced?
• In your opinion, what are the main consequences of iodine/Vitamin A deficiency?
• In your opinion, what percentage of households in Bangladesh consume fortified salt/oil?
• What do you think is likely to happen to fortified salt/oil during transportation, storage, and distribution?
• Do you know what is the name of the new body that the Ministry of Industries is planning to establish in order to ensure standardisation, regulatory monitoring, QA/QC [quality assurance/quality control] of all fortified food and products?

A set of options was provided with each question, and respondents had to choose among them, but only one of the options was right.^11^ Among oil firms, the correct responses were given by 77%, 89%, 20%, 69%, and 6% of them, for the first, second, third, fourth and fifth question respectively. Across the same questions, percentage of salt firms that gave correct responses were 64%, 98%. 23%, 6%, and 12% respectively. These questions did not just test the respondents’ understanding of the normative environment, but also examined broader aspects related to the fortification policy, such as familiarity with the health implications of insufficient Vitamin A/iodine.^12^ Thus, it tries to assess if the firm has internalised not just the regulatory framework, but a broader set of implications surrounding food fortification. The number of correct answers forms our engagement score [*Engagement*], which thus lies in the interval [0,5].

*Inspections*: To capture monitoring and enforcement at the firm level we measure the frequency of inspections by government authorities per year. Firms were asked about the number of inspections that they have per year.

*Effectiveness:* The perceived effectiveness of the institutional devices set by the government was gauged through a different section of the survey concerning the incentives and penalties already in place. Firms were asked if they knew of any incentive/penalty enforced by the authorities, their type, and the firm’s perception of the effectiveness of each category. This last feature was evaluated on a scale ranging from *Not at all effective* to *Very effective*, mapped into the interval [1,5]. An arbitrary value of 0 was assigned to firms that were not aware of any incentive/penalty set by the government. We then averaged across the different measures to obtain a proxy of the perceived effectiveness of the institutional regime [*Effectiveness*].

##### 4.1.2.3 Control variables

To control for potential confounders that might be relevant in our setting, we also include a series of controls that are meant to capture basic firms’ characteristics and additional features of the institutional environment. Control variables include: a proxy for firm size [*Employees*] – number of workers employed by the firm in 2018; the number of years since the establishment started operations [*Age*]; and the firm’s reported average degree of importance (on a scale from 1-Most important to 9-Least important) across three out of nine institutional factors that require improvement to ensure compliance with fortification standards (i.e., clear regulations, regulatory agency structure, and capacity) [*AvgInst*].

#### 4.1.3 Empirical strategy

Our analysis aims to understand the factors explaining compliance with oil and salt fortification regulations. Depending on the nature of the dependent variable, we employ different econometric models – logit for binary variables and generalised linear modelling (GLM) for fractional variables. We estimate the following:

(1) $${\mathrm{Comply}}_{i}={f(\beta }_{0}+\beta_{1}{\mathrm{Engagement}}_{i}+\beta_{2}{\mathrm{Inspections}}_{i}+\beta_{3}{\mathrm{Effectiveness}}_{i}+x_{i}\gamma )$$

where Complytakes the value of 1 if the firm is complying effectively with fortification regulations and 0 otherwise, Engagement is a measure of firms’ knowledge in relation to food fortification; Inspectionsreflects a measure of frequency of audits; Effectiveness captures the perceived effectiveness of incentives and penalties and x is a vector of control variables that may affect compliance at the firm level.

We adopt robustness checks to examine the structural validity of the analysis. First, we make use of the discrete CBS 1 itself to re-estimate the baseline models to examine the validity of the main results. Second, we test the robustness of the findings using the alternative definition of the score (CBS 2). In addition, to address potential concerns about the results with a small sample size for the oil firms and to support the main insights, we use bootstrapping^13^ to validate the model building process for the oil sample.

Finally, we also use principal component analysis (PCA) to assess the robustness of CBS 1′s construction procedure. From our methodology, it is clear that the different levels of assessment (i.e. use, source, access and quality) carry equal weight within the score. To test this assumption, we rely on PCA by using the loadings of the first component (the one that explains most of the variance in the underlying variables) as weights for the different elements of CBS 1. We call the resulting score the *Compliance Index (CI)* which is then normalized in the interval [0,1] and used to create a dummy variable for compliance when the index is higher than the average (to make the procedure equivalent to the one employed for the original CBS 1). We then rerun all our estimations using the dummy based on the index instead. In Table A2 in Appendix C we report the first eigenvector that we obtain from the PCA and the proportion of variance that it captures, for both the sample of oil and salt firms.

### 4.2 Qualitative data

In addition to the quantitative data, we obtained qualitative data based on in-depth qualitative interviews with key stakeholders between August and October 2019. In our qualitative inquiry, we gathered insights on the challenges of fortification by oil and salt firms, patterns of inspection, and the regulatory environments of inspection. Participants were selected, using stratified purposeful sampling method (Patton, 2002), to capture diversity of views of individuals who are considered ‘typical’ of their roles along the value chains. The qualitative insights complement the quantitative analysis in contextualising our regression results.

For the oil sector, we interviewed a total of 10 edible oil firms, 6 wholesalers, 8 retailers, one representative from the packers’ association, one premix supplier. For the salt sector, we interviewed 10 salt firms, 8 wholesalers, 11 retailers, and 3 representatives from salt mills associations. In addition, we conducted 5 and 3 focus group discussions (FGDs) with oil and salt consumers, respectively. These interviews addressed challenges of fortification, market incentives for fortification by the firms and the patterns of regulation enforcement along the two value chains. Moreover, to obtain information about the regulatory environment and challenges of law enforcement, we conducted further 5 interviews with key informants (Nutrition International, Scaling Up Nutrition (SUN) Business Network, GAIN Bangladesh, the Ministry of Industries (MoInd), and Bangladesh Small and Cottage industries Corporation (BSCIC)).

## 5 Results

To gain a better understanding of how differences in the main variables of interest are systematically related to fortification standards, we compare complying and non-complying firms. We first show the potential pitfalls of using standard measures of compliance (both formal and reported). Next we move to characterising the differences between complying and non-complying firms as defined by our main score, using the dummy variable Comply. Then, we describe the findings from our empirical models exploring the factor explaining firms’ compliance with food fortification standards in the Bangladeshi context, *ceteris paribus*.^14^ Given the idiosyncrasies that shape the institutional environment of salt and oil industries and, in particular, the different timing of when mandatory fortification was institutionalised, we conduct our analysis for each sample of firms separately.

### 5.1 Descriptive analysis

The edible oil producers in our sample produce soybean oil and palm oil (including super palm oil), which are the most widely consumed edible oils in Bangladesh, and which are considered fortifiable. Both oil refineries and oil packers procure Vitamin A premix themselves and fortify oil before packaging. This study examines refineries and packers as separate groups and then models their compliance behaviour as one group. The salt producers in our sample produce fine-grain, medium-grain, and non-refined salt as main products – covering edible and industrial salt, as the distinction between these two remains unclear.

The sample of oil and salt firms surveyed reported very high compliance, using both formal and reported measures. Regarding formal compliance, oil firms were asked: *‘Have you signed the MoU with the Ministry of Industry?*’ 74% of oil firms reported formal compliance. Reported compliance was queried directly, asking ‘*Are you using any Vitamin A in your product?*’, and interestingly, the same oil sample reported 91% compliance. Salt firms were asked about formal and reported compliance, in response to the following questions, respectively: ‘*Have you submitted a report to BSCIC regarding fortification status in the last year?*’, and ‘*Are you using any iodine in your product?*’ salt firms reported 97% and 90% formal and reported compliance respectively.

These high levels of formal and reported compliance are consistent with firm’s reluctance in reporting during the pilot study, and are therefore unlikely to help study the factors explaining compliance among firms, given their low variability. And while every effort was made to collect information on actual compliance at the firm-level, it proved impossible to gather product samples within the scope of this study. In fact, during our pilot survey, we discovered that there are numerous gaps in collecting and testing samples in-country. To overcome these limitations, we designed a survey that focused on the process of fortification.

We rely on our CBS 1 score to gain a better understanding of compliance behaviour among firms. Table 3 reports the average CBS 1 score for oil and salt firms. Salt firms on average score 0.618 on compliance, and oil firms score higher than salt firms and are at an average of 0.753.^15^ The difference in the mean value of CBS 1 between the two samples of firms is statistically significant (t=-2.6049,p=0.0102).

**Table 3:** Compliance score for oil and salt firms.

CBS1	Mean	Standard deviation	Min.	Max.
OIL (35)	0.753	0.276	0.141	0.997
SALT (102)	0.618	0.261	0.000	0.994

**Fig. A3:**
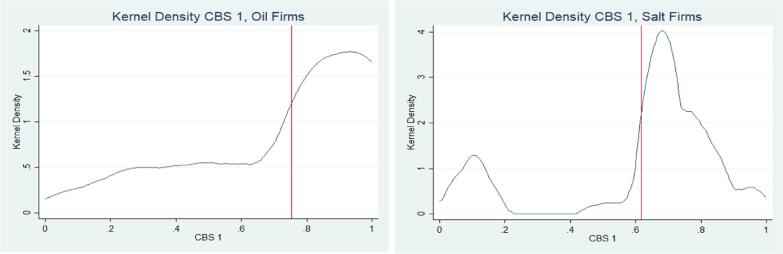
Distribution of CBS 1, by type of firm. NOTE: Kernel density visualises the distribution of data over the score. Red line shows the mean score. (For interpretation of the references to colour in this figure legend, the reader is referred to the web version of this article.)

**Fig. A4:**
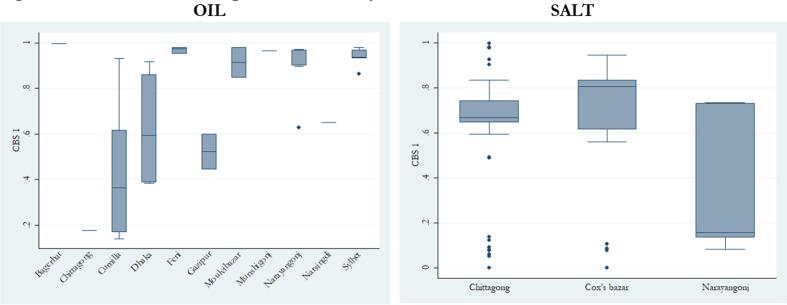
Box-and-whisker plots for CBS 1 by districts.

The distribution of CBS 1 for oil and salt producers (Fig. A3in Appendix B) reveals that the probability of achieving a better compliance score is higher for oil firms, while this is much more varied for salt firms as we note a bimodal distribution. When we examine the distribution of CBS 1 by districts (Fig. A4in Appendix B) we also note some differences. For instance, looking at the sample of salt firms we see that those that are based in the district of Narayangonj have a median score that is more than 0.5 points lower than the others.

Next, we compared the key variables from the quantitative survey with the qualitative data that generally affirmed the descriptive results. We discuss two real-life examples here. First, looking at oil firms, a good example emerges from one oil refinery with its main factory in Narayangonj that stressed the extremely high price of premix as the main challenge for fortification, in addition to reporting no specific business benefits coming from fortification and that the procedure was carried out only to comply with regulations. In addition, we found that the respondent lacked knowledge of the fortification process and only mentioned temporary bans as the main risk of not fortifying food. Comparing with our survey data, we find this reflected in our CBS 1 score for the firm (0.385, within the 1st quartile of the score) and its “engagement” (2, within the 1st quartile of the variable).

Second, when we look at salt firms, one firm, also based in Narayangonj, claim that they started fortifying the product even before it became mandatory. The firm asserted that the procedure does not significantly increase the cost of production and that non-complying firms should be punished as fortification is a “must” for businesses operating in this industry. Moreover, the respondent appeared to be quite knowledgeable about the fortification process, and the firm had proper chemists and a lab to control the whole procedure. In this case, the main risk of not fortifying was highlighted as the loss of the firm’s good reputation. Again, these qualitative insights are reflected in our quantitative measures: the values of CBS 1 and “engagement” are the highest among the sample of salt firms (0.994 and 4, respectively).

Further, to assess how the main variables of interest are systematically related to fortification standards, we analyse the differences between complying and non-complying firms, as defined by the dummy variableComply. Table 4 outlines key descriptive statistics for the sample of oil firms (*Panel A*) and salt firms (*Panel B*) reporting the mean values and standard deviations (in brackets) of the variables in the baseline estimations to follow. T-tests for the differences in the means between the complying and non-complying group are also reported. For the sample of oil firms, we observe a significant difference in the engagement score between complying and non-complying firms (p < 0.01), and in the perceived effectiveness of the penalties and incentives set by the government (at the 5% level). There is also a significant difference (at the 5% level) in food fortification engagement between the two subsamples for salt firms.

**Table 4:** Differences between complying and non-complying firms.

*Panel A: Oil sample*				
	All firms	Non-complying	Complying	T-test (non-com)
Engagement	2.600 (0. 945)	1.917 (0.900)	2.956 (0.767)	-3.587*** [0.001]
Inspections	2.686 (3.151)	2.167 (3.271)	2.956 (3.126)	-0.699 [0.490]
Effectiveness	3.005 (1.180)	2.542 (0.542)	3.246 (1.352)	-2.186** [0.036]
Employees	72.057 (196.334)	28.833 (39.177)	94.609 (239.279)	-0.939 [0.354]
Age	10.000 (8.708)	6.833 (6.177)	11.652 (9.475)	-1.588 [0.122]
AvgInst	3.324 (1.219)	3.167 (1.141)	3.406 (1.275)	-0.545 [0.589]
Observations	**35**	**12**	**23**	

*Panel B: Salt sample*				
Engagement	2.020 (0.770)	1.696 (0.822)	2.114 (0.733)	-2.341** [0.021]
Inspections	18.608 (11.012)	21.391 (12.837)	17.797 (10.373)	1.231 [0.228]
Effectiveness	3.421 (1.561)	3.373 (1.879)	3.436 (1.469)	-0.168 [0.867]
Employees	53.951 (40.271)	43.391 (16.450)	57.025 (44.511)	-1.436 [0.154]
Age	23.000 (15.046)	17.783 (10.867)	24.519 (15.794)	-2.339** [0.023]
AvgInst	3.000 (1.096)	2.855 (0. 973)	3.042 (1.131)	-0.719 [0.474]
Observations	**102**	**23**	**79**	

Note: Standard deviations are reported in parentheses. P-values are reported in square brackets. We select the appropriate t-test by first testing for equality of variances between the two groups using a (Icddr’b, 2015) robust statistic (Brown and Forsythe, 1974) *** p < 0.01, ** p < 0.05, * p < 0.1.

### 5.2 Oil firms

Table 5 reports the baseline results for the oil sample using logit estimation, where we estimate equation [1] by sequentially adding the primary variables of interest [Models I–V]. Column (I) includes Engagementand all control variables; columns (II) and (III) introduce Inspections and Effectiveness in turn; column (IV) replaces the raw number of inspections for an inspection dummy that captures higher frequency of inspections on average; and column (V) examines the robustness of the baseline by bootstrapping using five replications of the sample to validate the model building process (rather than estimates).

**Table 5:** Explaining compliance – oil firms.

Dependent variable: Dummy *Comply*					
Variables	(1)	(2)	(3)	(4)	(5)
	Model I	Model II	Model III	Model IV	Model V
	*Baseline*	*Inspections, Incentives & Penalties*			*Bootstrapping*
Engagement	1.571***	1.533***	1.221*	1.594**	1.571***
	**(0.504)**	**(0.515)**	**(0.654)**	**(0.748)**	**(0.534)**
*Inspections*					
Inspections		−0.095 (0.305)	−0.081 (0.299)		
Insp dummy				2.040 (1.676)	
*Incentives and penalties*					
Effectiveness			0.482	0.717	
			(0.824)	(0.587)	
*Controls*					
Employees	0.001	0.001	0.001	−0.001	0.001
	(0.001)	(0.002)	(0.002)	(0.002)	(0.019)
Age	0.050	0.073	0.102	0.069	0.050
	(0.108)	(0.084)	(0.129)	(0.156)	(0.118)
AvgInst	0.258	0.203	0.203	0.376	0.258
	(0.452)	(0.400)	(0.385)	(0.514)	(0.784)
Constant	−4.558**	−4.286**	−5.251	−7.632**	−4.558
	(1.907)	(1.995)	(3.244)	(3.400)	(3.030)
**Observations**	**35**	**35**	**35**	**35**	**35**
**Pseudo R-Square**	**0.287**	**0.290**	**0.313**	**0.359**	**0.287**

Robust standard errors in parentheses. *** p < 0.01, ** p < 0.05, * p < 0.1.

We find a positive and significant relationship between Engagement and compliance across all five models in Table 5, suggesting that greater engagement with food fortification including knowledge around the issues surrounding fortification and its regulation, as well as the health implications and current consumption levels of fortified food, is associated with greater firms’ compliance, providing strong evidence in favour of **H1**. Using Model IV, the coefficient for Engagement implies that, *ceteris paribus*, an increase of one point in the score on average is predicted to increase the probability of complying by 31.4 percentage points.^16^

We find no evidence for **H2**, there being no significant correlation between compliance and the frequency of inspections for oil firms in Models II–III in Table 5**.** To examine the implications of the results for **H2** closely, we consider the possibility that inspections by regulatory authorities are effective only when they are more frequent than usual. So, in Model IV, we introduce a dummy that takes a value of 1 if the number of regulatory audits is higher than the average, and 0 otherwise, to compare firms that are inspected the most with those that are inspected less frequently. We note a positive (**H2**) but still weak correlation with compliance. In Models III and IV, Effectiveness has the expected positive sign (in line with **H3**), but its effect is not statistically different from 0.

The non-significant effect of Inspections and Effectiveness may owe to the more recent introduction of fortification regulation for edible oil in 2013 (Aaron et al., 2017), such that firms are yet to be accustomed to all the necessary requirements for effective compliance for inspections to have any clear effects. The positive correlation for the group inspected with a greater frequency also suggests the same.

Further, our qualitative data point to some inconsistencies in regulatory enforcement in the oil sector. More specifically, large-scale oil firms, who produce traceable bottled oil and untraceable bulk oil products, are regulated through compliance inspections at the retail and wholesale level. In other words, they do not receive regulators at firm-gate but regulators can trace the origin of bottled oil to specific firms and enforce the fortification status through this channel. In contrast, small- and medium-scale firms are inspected for compliance at the firm-gate. Inspections likely capture both of these two mechanisms. However, the former – i.e. traceability-based mechanism – incentivises firms to fortify bottled – i.e. traceable – oil products but not bulk. Therefore, the non-significant effect of Inspections may also be attributed to the fact that compliance inspections do not affect bulk oil products, which are often inadequately fortified (GAIN and icddr’b, 2017). Additionally, inspections may be for reasons beyond compliance and include inspection of equipment as opposed to actually taking a sample and testing it for Vitamin A.

### 5.3 Salt firms

Table 6 reports the results for the salt sample using logit estimation, presenting models by sequentially adding the primary variables of interest [Models I–V]. The first four columns reproduce the specifications used for the sample of oil firms. Column (I) includes Engagement with all control variables; columns (II) and (III) introduce Inspections and Effectiveness in turn; and column (IV) replaces the number of inspections with the inspection dummy that captures higher frequency of inspections on average.

**Table 6:** Explaining compliance – salt firms.

Dependent Variable: Dummy *Comply*					
Variables	(1)	(2)	(3)	(4)	(5)
	Model I	Model II	Model III	Model IV	Model V
	*Baseline*	*Inspections, Incentives & Penalties*			*Interaction*
Engagement	0.743*	0.963**	0.932**	0.997**	0.166
	**(0.383)**	**(0.418)**	**(0.417)**	**(0.435)**	(0.637)
*Inspections*					
Inspections		−0.044*	−0.056**		−0.030
		**(0.023)**	**(0.027)**		(0.024)
Insp dummy				−1.537**	
				**(0.715)**	
*Incentives and penalties*					
Effectiveness			0.175	0.204	
			(0.199)	(0.200)	
Distrust					−3.106*
					**(1.648)**
*Interaction*					
Distrust*Engagement					1.991**
					**(0.915)**
*Controls*					
Employees	0.020	0.019	0.019	0.016	0.019
	(0.018)	(0.020)	(0.020)	(0.020)	(0.019)
Age	0.028*	0.032*	0.032**	0.030**	0.025
	(0.015)	(0.016)	(0.016)	(0.015)	(0.016)
AvgInst	0.261	0.243	0.212	0.149	0.147
	(0.198)	(0.194)	(0.204)	(0.224)	(0.238)
Constant	−2.497*	−1.986	−2.229	−2.530	−0.434
	(1.329)	(1.481)	(1.556)	(1.575)	(1.954)
**Observations**	**102**	**102**	**102**	**102**	**102**
**Pseudo R-Square**	**0.109**	**0.140**	**0.147**	**0.160**	**0.190**

Robust standard errors in parentheses. *** p < 0.01, ** p < 0.05, * p < 0.1.

Again, we find a positive and significant relationship between food fortification engagement and compliance across all five models in **6**, suggesting that, *ceteris paribus*, greater engagement with issues surrounding fortification and its regulation, as well as the health implications and current consumption levels of fortified food, is associated with greater firms’ compliance, providing strong evidence in favour of **H1**. The effect of food fortification engagement is also quite sizeable: on average, and *ceteris paribus*, an increase of one point is predicted to increase the probability of complying by 13.7 percentage points.

For the salt estimation, *ceteris paribus*, we find that the coefficient for Inspections is negative and significant in **6**, and the magnitude of the effect increases between Models II and III. The negative effect is again opposed to the expected hypothesised effect in **H2** (i.e., that compliance is likely to increase with more frequent inspections). In Model IV, we substitute the number of inspections with the dummy that captures inspections that are more frequent than the average, similarly to the sample of oil firms. However, interestingly, the effect stays negative and significant, in opposition to **H2**. Using Model IV as the baseline, the coefficient for inspection dummy implies that *ceteris paribus*, on average, being subject to a higher number of inspections reduces the probability of complying by 24.3 percentage points. These results seem to hint that firms that are more likely to be violators are inspected more often, as also suggested by Gupta et al. (2019). Indeed, our qualitative data shows that BSCIC, the regulatory body responsible for inspecting salt mills in Bangladesh, works closely with small- and medium-scale mills that tend to lack adequate facilities to conduct and monitor fortification. As a result, they inspect large-scale mills, i.e. compliant firms, less often than small- and medium-scale mills. In Models III and IV, in **6**, the perceived effectiveness of incentives and penalties has the positive predicted sign (**H3**), but it is again not statistically significant.^17^

We examine an additional hypothesis for incentives and penalties (**H4**) in the context of the salt industry in Bangladesh – the mediating effect of food fortification engagement on a firm’s distrust of incentives and penalties. Thus, engagement would diminish the adverse effects of a perceived ineffectiveness of enforcement measures. In Model V, we replace Effectiveness with a dummy variable Distrust that takes the value 1 for firms where average perceived effectiveness of the incentives and penalties set by the government is less than the 4.5.^18^ The negative sign on Distrust hints that weak regulatory enforcement has a detrimental effect on firm’s behaviour, as suggested by Luthringer et al. (2015). This is also in line with the insights from the qualitative data. Firstly, the fine that would be imposed if firms are found non-compliant by BSCIC is low (i.e. 50,000BDT or approximately 590 USD), providing limited incentives for firms to comply. Since the fortification law was introduced in 1989, the legal fee has not been updated. Secondly, BSCIC provides iodine premix to salt firms in Bangladesh and salt mills associations and one mill indicated that the delivery of iodine premix is often inadequate and/or late. This may lead to a situation where BSCIC is unwilling to punish mills for its own shortcomings in supplying premix at an adequate quantity within a given time frame.

**Fig. 3:**
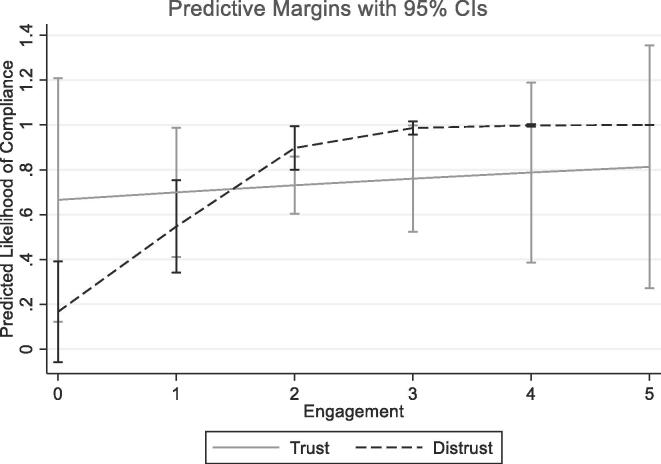
Predicted compliance for salt firms, by distrust (1) and trust (0). Note: Graph plots predicted compliance and confidence intervals by Distrust dummy. Predicted compliance on Y-axis. Engagement on X-axis. The difference in predictive margins of compliance, and its changing pattern with increasing engagement, affirms the role played by food fortification engagement.

To examine the effect of food fortification engagement in moderating this distrust, we include the interaction term Distrust×Engagement. We observe an expected positive and statistically significant effect for the interaction, which counterbalances the negative effect of the dummy Distrust, affirming the role of food fortification engagement in mediating the effect of distrust on compliance. Fig. 3 below plots the predicted likelihood of compliance for salt firms – by distrust (1) and trust (0) – that further depicts the mediating role of Engagement. Even for firms that generally distrust incentives and penalties (distrust (1)), a greater engagement is linked with an increase in compliance.

### 5.4 Robustness

To assess the reliability of our findings we perform a series of robustness checks. First, we replicate our results employing the discrete CBS 1 itself instead of the dummy Comply.^19^ We then re-estimate the baseline specifications using the alternative definition of the score (CBS 2). The results from these two robustness checks are in Appendix D, Appendix E.

We also compute an alternate *Compliance Index* (CI) using principal components analysis (PCA). The estimates obtained when the dependent variable is the compliance dummy based on the index computed through the PCA is reported in Appendix F.

Results for the oil sample are unaffected by the changes in the variable underlying the dummy.^20^ When we look at the salt sample, the negative effect of Inspectionspersists and the magnitude of the coefficients for Engagement is slightly smaller across the models. However, the main insights that we get are the same as those from the baseline estimations.

Overall, the above analysis together with the ones reported in the Appendix suggest that our previous results are robust and unaffected. The results for Engagement appear to be the most robust and consistent across the different checks. In addition, the mediating effect of food fortification engagement we captured in our baseline estimations is quite robust as well.

### 5.5 Validation

We examine the predictive power of our compliance score relative to government standards on micronutrient content for food fortification in Bangladesh.^21^ Using reported total yearly production and the type and amount of fortificant used by the oil and salt firms, we obtain an estimate of the average concentration (in part per million or ppm) of micronutrient in the final product. Next, we create a dummy variable ‘*WithinStd*’ that takes the value of 1 if the estimate of the micronutrient concentration falls within the government’s standard and 0 otherwise.^22^ Finally, we run logit models (Appendix G) using *WithinStd* as dependent variable, and our CBS1 score as the main covariate with the control variables. We find a positive and statistically significant coefficient for CBS1 both for oil and salt firms, which implies that an increase in our compliance score is correlated with the firms reporting more accurate fortification.

## 6 Discussion and policy implications

Regulatory monitoring is conducted by national inspectorate agencies at the levels of production sites borders, customs warehouses or retail stores to confirm that food is fortified at levels that comply with relevant legislation and standards. At firm production sites, such an inspection visit comprises two broad categories of activities. First, an audit that includes verification, inspection, and audits of quality assurance and quality control (QA/QC) processes and fortification inputs such as micronutrient premix. This component involves the inspector completing an audit checklist to ascertain quality management: confirmation that processes, systems and equipment are in place that ensure the production of a quality product. Second, product sampling and testing of composite samples for nutrient content.

Tackling this challenge of measuring conformity to micronutrient specifications in nationally adopted food fortification standards head on, the unique CBS score provides an alternative to the more prohibitive quantitative tests that often result in weaknesses in regulatory monitoring. Our methodology can be incorporated into current practice by building on the existing audit checklist and adding a theory-based metric of compliance behaviour, the CBS score. In fact, this method is significantly cheaper to implement in comparison of the costs of lab testing, providing cost savings of 30%-40% relative to product sampling and analysis for nutrient content.

We find that greater engagement with regulations is significantly linked to firms’ compliance with food fortification. This refers to the likely benefits from improving firm’s knowledge around the issues surrounding fortification and its regulation, as well as the health implications and current consumption levels of fortified food – as in the case of oil and salt in Bangladesh. This finding is similar to UK’s HSE commissioned research^23^ which identifies that a need for education and awareness remains when promoting compliance to regulations. Our study finds that returns from investing in improving engagement of oil and salt firms in Bangladesh is also quite sizable.

In fact, engagement with fortification can also diminish the negative effect of a general distrust of government effectiveness in deterring non-compliance. For example, salt firms in Bangladesh only incur a very low fine for non-compliance that has not been updated since its introduction in 1989. In such a scenario, implementation of programmes targeted at increasing food fortification engagement can be a useful instrument to improve firms’ likelihood of compliance, especially in the salt industry. This would not just facilitate the implementation of food fortification, but would also help mitigate the adverse impact of distrust in incentives and penalties for salt firms operating in a complex economic setting such as Bangladesh.

Looking to institutional factors, there appear to be some inconsistencies in regulatory enforcement, as seen in the oil sector. While large-scale oil firms that produce a combination of traceable bottled oil and untraceable bulk oil products in Bangladesh are regulated through compliance inspections at the retail and wholesale level, small- and medium-scale firms are inspected for compliance at the firm-gate. The former may be incentivising firms to fortify bottled oil products but the same may not be true for bulk oil.

Further, we find that inspections may work for some firms (for oil firms in Bangladesh) only when they occur with greater frequency i.e., they occur more often in a given year. This finding may be partly due to the recent introduction of the regulation for oil firms in Bangladesh, which could demand persistent regulatory audits to ensure that firms get accustomed to all the necessary requirements for effective compliance. But it also affirms the positive correlation between inspections and compliance as in Hawkins (2010). Overall, this result suggests that in an institutional environment that has experienced changes, government inspections could reduce the perceived cost of compliance by, for instance, helping firms to assess the quality of the new fortification equipment. The result also suggests that there are indirect benefits from inspections – especially towards improvements in quality of final product, once equipment and processes are assessed.

However, for other firms (salt firms in Bangladesh), we find that being subject to greater inspections is associated with lower compliance. A likely explanation for this is that in the given institutional environment, the greater frequency of government inspections might be systematically related to poorer performance– suggesting that more regular audits tend to target those firms that are more likely not to comply with fortification regulations. In fact, our qualitative data suggests that BSCIC, the regulatory body responsible for inspecting salt mills in Bangladesh, inspects large-scale mills that also tend to be more compliant firms, less often than small- and medium-scale mills. It may also be the case that for salt firms operating within a more mature and consolidated institutional framework, frequent inspections are perceived only as an additional burden by complying firms, such that we find greater inspections are linked with lower compliance.

Finally, there are two directions of future research that emerge from our study. First, full external validity of the methodology and score against product testing at a disaggregated level. Second, we aim for our compliance measure to feed into a sustainable longer-term compliance method combining frequent monitoring using the compliance behaviour score with occasional quantitative product testing.

## 7 Conclusion

The compliance score produced in this study presents a unique framework for measuring/predicting compliance and identifying significant factors explaining compliance that can be used to inform modifications in fortification programmes and policy. This tool captures compliance based on different stages of a standard compliance process following a firm’s decision to comply (Henson and Heasman, 1998). We apply the methodology for fortification standards for oil and salt producers in Bangladesh. In doing so, this study presents, to the best of our knowledge, the first systematic empirical evidence on the topic.

First, we find that engagement is significantly linked with compliance, pointing towards prioritizing greater investments in education and awareness when promoting compliance to regulations. Second, we find that greater frequency of face-to-face interactions can improve compliance in the case of recent introduction of the regulation. Third, firms’ distrust in the effectiveness of incentives and penalties can hamper compliance, but when firm are more aware and engaged, they have a better understanding of the policy that helps recognize the value of complying for their brand.

In summary, compliance and enforcement strategies that combine increasing engagement with a prudent inspections policy that accounts for different institutional settings are more likely to enhance compliance behaviour for oil and salt firms in Bangladesh. Prioritising future programmes that enable such mechanisms should be priority for governments and other stakeholders working to improve compliance to food fortification standards.

## Credit authorship contribution statement

**Amrita Saha:** Conceptualization, Methodology, Data curation, Formal analysis, Project administration, Validation, Funding acquisition, Writing – original draft, Supervision. **Daniele Guariso:** Methodology, Data curation, Formal analysis, Writing – original draft & editing. **Mduduzi N.N. Mbuya:** Visualization, Funding acquisition, Writing – review & editing. **Ayako Ebata:** Conceptualization, Writing – original draft.

## Declaration of Competing Interest

The authors declare that they have no known competing financial interests or personal relationships that could have appeared to influence the work reported in this paper.

## Figures and Tables

**Table A1:** Components underlying compliance score, by type of firm.

Oil firms	Salt firms	Description
**Storage facility for premix**	**Storage facility for premix**	Facility to store premix obtained by firm – these can be inventories or simple storage in warehouse.
**Premix measurement equipment**	**Premix measurement equipment**	Apparatus to accurately measure premix – salt or oil premix needs differ for equipment to facilitate measurement of iodine/Vitamin A.
**Mixers**	**Mixers**	Mixers can be of different types depending on scale, for example, small, electric, rotating-drum, etc.
**Fortification related IT use**	**Fortification related IT use**	Any information technology needs to fortify or better inform the process of fortification.
**Testing samples (lab facilities)** a. Single ‘grab sample’ b. Composite sample	**Testing samples (lab facilities)** a. Single ‘grab sample’ b. Composite sample	Laboratory facilities to test samples of oil or salt for adequate fortification and retention.
**Recording (of premix, mixing, final products, etc.)** a. Premix stocktake b. Premix calculations c. Equipment maintenance d. Equipment calibration e. Mixing procedure f. Frequency of sampling g. Point of sampling h. Test methods validation i. Corrective actions	**Recording (of premix, mixing, final products, etc.)** a. Premix stocktake b. Premix calculations c. Equipment maintenance d. Equipment calibration e. Mixing procedure f. Frequency of sampling g. Point of sampling h. Test methods validation i. Corrective actions	Records on stock-taking should reflect the verification of the quantities and condition of premix held in inventory or warehouse. Records on premix calculations refers to the addition formulae used. Records on equipment maintenance should reflect the routine checks on the fortification equipment. Records on equipment calibration refers to setting the appropriate rate of delivery of the micronutrient within the defined range. Records on the mixing procedure should show that the premix delivery system has been validated. Records on the frequency of sampling should reflect that the frequency is appropriate for the final quantity of product produced. Records on the point of sampling should show that samples or sub-samples are taken from predetermined point or points in the production process. Records on test methods validation should ensure that the testing process is suitable for its intended use. Records on corrective actions should show the interventions taken if verification procedures confirm a product is potentially out-of-specification.
**Titration equipment**		Titration equipment specific to oil firms to determine the concentration of a substance in a solution.
**Blenders**		Blenders needed for oil firms to mix Vitamin A.
	**Spraying technology**	Spraying is an important part of salt fortification as it involves spraying a solution that contains iodine.
	**Salt Iodization Plant**	Salt iodization plants facilitate the procedure of fortification.
	**Salt test kits**	Kits to test salt for requisite levels of iodine.

**Table A2:** First eigenvector and proportion of variance that explains, by type of firm.

Principal Component Analysis		
	Oil Sample	Salt Sample
Proportion of Variance Explained	96%	72%
**Loadings**		
Use	0.505	0.580
Source	0.505	0.273
Access	0.505	0.580
Quality	0.484	0.502

**Table A3:** CBS1 - Oil firms.

Dependent Variable: CBS 1-discrete				
	(1)	(2)	(3)	(4)
Variables	Model I	Model II	Model III	Model IV
	*Baseline*	*Inspections, Incentives & Penalties*		
Engagement	0.582** (0.227)	0.524** (0.230)	0.370 (0.309)	0.470* (0.263)

*Inspections*				
Inspections		−0.137 (0.163)	−0.118 (0.149)	
Insp dummy				1.061 (0.713)
*Incentives and penalties*				
Effectiveness			0.266 (0.310)	0.434* (0.248)
		
*Controls*				
Employees	0.001 (0.001)	0.002 (0.002)	0.002 (0.001)	0.000 (0.001)

Age	0.010 (0.052)	0.045 (0.040)	0.054 (0.046)	0.012 (0.056)

AvgInst	0.031 (0.277)	−0.050 (0.218)	−0.055 (0.207)	0.057 (0.260)

Constant	−0.580 (0.905)	−0.187 (0.807)	−0.697 (0.992)	−1.826** (0.825)

**Observations**	**35**	**35**	**35**	**35**

Robust standard errors in parentheses. *** p < 0.01, ** p < 0.05, * p < 0.1.

**Table A4:** CBS1 - Salt firms.

Dependent Variable: CBS 1-discrete					
Variables	(1)	(2)	(3)	(4)	(5)
	Model I	Model II	Model III	Model IV	Model V
	*Baseline*	*Inspections, Incentives & Penalties*			*Interaction*
Engagement	0.244 (0.149)	0.309** (0.144)	0.305** (0.141)	0.299** (0.142)	−0.157 (0.261)
*Inspections*					
Inspections		−0.019* (0.010)	−0.023** (0.011)		−0.013 (0.012)
		
Insp dummy				−0.496* (0.259)	
				
*Incentives and penalties*					
Effectiveness			0.058 (0.079)	0.051 (0.080)	
			
Distrust					−1.480** (0.684)
*Interaction*					
Distrust*Engagement					0.818** (0.333)
*Controls*					
Employees	0.010** (0.005)	0.009* (0.005)	0.009* (0.005)	0.009* (0.005)	0.008 (0.005)

Age	0.008 (0.006)	0.009 (0.007)	0.009 (0.006)	0.008 (0.006)	0.005 (0.006)

AvgInst	0.124* (0.065)	0.106 (0.068)	0.098 (0.070)	0.083 (0.073)	0.017 (0.081)

Constant	−1.064** (0.511)	−0.734 (0.543)	−0.840 (0.570)	−0.971* (0.561)	0.411 (0.775)

**Observations**	**102**	**102**	**102**	**102**	**102**

Robust standard errors in parentheses. *** p < 0.01, ** p < 0.05, * p < 0.1.

**Table A5:** CBS2 - Oil firms.

Dependent variable: Dummy Comply based on CBS 2				
Variables	(1)	(2)	(3)	(4)
	Model I	Model II	Model III	Model IV
	*Baseline*	*Inspections, Incentives & Penalties*		
Engagement	1.571*** (0.504)	1.533*** (0.515)	1.221* (0.654)	1.594** (0.748)

*Inspections*				
Inspections		−0.095 (0.305)	−0.081 (0.299)	
		
Insp dummy				2.040 (1.676)
			
*Incentives and penalties*				
Effectiveness			0.482 (0.824)	0.717 (0.587)
		
*Controls*				
Employees	0.001 (0.001)	0.001 (0.002)	0.001 (0.002)	−0.001 (0.002)

Age	0.050 (0.108)	0.073 (0.084)	0.102 (0.129)	0.069 (0.156)

AvgInst	0.258 (0.452)	0.203 (0.400)	0.203 (0.385)	0.376 (0.514)

Constant	−4.558** (1.907)	−4.286** (1.995)	−5.251 (3.244)	−7.632** (3.400)

**Observations**	**35**	**35**	**35**	**35**
**Pseudo R-Square**	**0.287**	**0.290**	**0.313**	**0.359**

Robust standard errors in parentheses. *** p < 0.01, ** p < 0.05, * p < 0.1.

**Table A6:** CBS2 - Salt firms.

Dependent variable: Dummy Comply based on CBS 2					
Variables	(1)	(2)	(3)	(4)	(5)
	Model (I)	Model (II)	Model (III)	Model (IV)	Model (V)
	*Baseline*	*Inspections, Incentives & Penalties*			*Interaction*
Engagement	0.611* (0.334)	0.708** (0.342)	0.699** (0.339)	0.720** (0.341)	−0.045 (0.564)

*Inspections*					
Inspections		−0.023 (0.020)	−0.029 (0.023)		−0.011 (0.022)
		
Insp dummy				−0.798 (0.577)	
				
*Incentives and penalties*					
Effectiveness			0.089 (0.171)	0.104 (0.172)	
			
Distrust					−2.540* (1.455)
				
*Interaction*					
Distrust*Engagement					1.518** (0.730)
				
*Controls*					
Employees	0.018 (0.014)	0.016 (0.014)	0.016 (0.014)	0.015 (0.014)	0.016 (0.014)

Age	0.023 (0.015)	0.024 (0.015)	0.025*	0.024 (0.015)	0.019 (0.015)
			(0.015)		
AvgInst	−0.027 (0.223)	−0.043 (0.232)	−0.059 (0.233)	−0.092 (0.243)	−0.175 (0.266)

Constant	−1.474 (1.138)	−1.135 (1.205)	−1.267 (1.245)	−1.415 (1.246)	0.544 (1.852)

**Observations**	**102**	**102**	**102**	**102**	**102**
**Pseudo R-Square**	**0.0864**	**0.0954**	**0.0976**	**0.101**	**0.131**

Robust standard errors in parentheses. *** p < 0.01, ** p < 0.05, * p < 0.1.

**Table A7:** Compliance Index – Oil firms.

Dependent variable: Dummy *Comply* based on CI				
Variables	(1)	(2)	(3)	(4)
	Model I	Model II	Model III	Model IV
	*Baseline*	*Inspections, Incentives & Penalties*		
Engagement	1.571***	1.533***	1.221*	1.594**
	**(0.504)**	**(0.515)**	**(0.654)**	**(0.748)**
*Inspections*				
Inspections		−0.095 (0.305)	−0.081 (0.299)	
Insp dummy				2.040 (1.676)
*Incentives and penalties*				
Effectiveness			0.482	0.717
			(0.824)	(0.587)
*Controls*				
Employees	0.001	0.001	0.001	−0.001
	(0.001)	(0.002)	(0.002)	(0.002)
Age	0.050	0.073	0.102	0.069
	(0.108)	(0.084)	(0.129)	(0.156)
AvgInst	0.258	0.203	0.203	0.376
	(0.452)	(0.400)	(0.385)	(0.514)
Constant	−4.558**	−4.286**	−5.251	−7.632**
	(1.907)	(1.995)	(3.244)	(3.400)
**Observations**	**35**	**35**	**35**	**35**
**Pseudo R-Square**	**0.287**	**0.290**	**0.313**	**0.359**

Robust standard errors in parentheses. *** p < 0.01, ** p < 0.05, * p < 0.1.

**Table A8:** Compliance Index – Salt firms.

Dependent Variable: Dummy *Comply* based on CI					
Variables	(1)	(2)	(3)	(4)	(5)
	Model I	Model II	Model III	Model IV	Model V
	*Baseline*	*Inspections, Incentives & Penalties*			*Interaction*
Engagement	0.548	0.848**	0.801**	0.864**	−0.219
	(0.362)	**(0.406)**	**(0.397)**	**(0.412)**	(0.610)
*Inspections*					
Inspections		−0.061**	−0.076**		−0.046*
		**(0.025)**	**(0.030)**		**(0.026)**
Insp dummy				−1.954**	
				**(0.772)**	
*Incentives and penalties*					
Effectiveness			0.228	0.248	
			(0.211)	(0.219)	
Distrust					−4.571***
					**(1.702)**
*Interaction*					
Distrust*Engagement					2.833***
					**(0.987)**
*Controls*					
Employees	0.017	0.014	0.014	0.010	0.014
	(0.016)	(0.019)	(0.020)	(0.018)	(0.019)
Age	0.030**	0.036**	0.037**	0.033**	0.027
	(0.015)	(0.018)	(0.017)	(0.016)	(0.018)
AvgInst	0.321*	0.309*	0.280	0.193	0.193
	(0.189)	(0.187)	(0.196)	(0.224)	(0.237)
Constant	−2.124*	−1.406	−1.744	−2.099	0.863
	(1.231)	(1.425)	(1.486)	(1.472)	(2.086)
**Observations**	**102**	**102**	**102**	**102**	**102**
**Pseudo R-Square**	**0.093**	**0.153**	**0.165**	**0.174**	**0.241**

Robust standard errors in parentheses. *** p < 0.01, ** p < 0.05, * p < 0.1.

**Table A9:** Logit models for oil and salt firms, using *WithinStd.*

	(1)	(2)
Variables	Oil	Salt
CBS1	4.646***	1.851**
	***(1.637)***	***(0.787)***
*Controls*		
Employees	−0.003*	0.001
	(0.001)	(0.010)
Age	0.168**	0.047***
	(0.073)	(0.017)
AvgInst	−0.203	−0.036
	(0.299)	(0.332)
Constant	−4.454**	−4.567***
	(1.862)	(1.490)

**Observations**	35	102
**Pseudo R-Square**	0.308	0.108

Robust standard errors in parentheses. *** p < 0.01, ** p < 0.05, * p < 0.1.

## Appendix A Compliance score details

See Table A1.

## Appendix B Additional descriptives

See Fig. A1, Fig. A2, Fig. A3, Fig. A4.

## Appendix C Principal component analysis

See Table A2.

## Appendix D Robustness Checks: CBS 1 – Discrete

See Table A3, Table A4.

## Appendix E Robustness Checks: CBS 2

See Table A5, Table A6.

## Appendix F Robustness Checks: Compliance Index

See Table A7, Table A8.

## Appendix G Validation

See Table A9.

## Footnotes

1 For example, a recent assessment of regulatory monitoring across different country programs revealed costs and capacity for laboratory testing to be prohibitive, with 38% of respondents reporting a lack of equipment and inputs for laboratories, and 50% indicating a lack of trained staff and technical capacity (Luthringer et al., 2015). Further, our own assessment from production monitoring data across 20 example sites (data available on request from authors) suggests that the process only including an audit would incur an indicative cost of USD 18,500, but when product sampling (5 samples from 2 batches per industry) and analysis for nutrient content (across a range of typical foods and nutrients) are included, additional costs of USD 18,000 are incurred.

2 We provide a comparison of costs in Section 6.

3 For instance in Bangladesh, the salt iodisation law remains unclear regarding enforcement of the law and the consequences of non-compliance (WHO, 2011). As a result, salt mills in Bangladesh are unable to measure the risk of non-compliance effectively.

4 Another study of flour fortification in Pakistan found that fortification interventions attempted to leverage public–private partnerships in a segment of the wheat flour value chain beset with regulatory weakness, suggesting that technical interventions should support rather than ignore a broader agenda of reforms in food policy (Ansari et al., 2018), also aligning with more recent findings highlighting realism about the contribution of major businesses to public nutrition objectives (Poole et al., 2020).

5 Firm’s perceived effectiveness of incentives and penalties will be captured by responses provided through a survey. The survey was targeted to firm managers and owners or a firm representative with sufficient knowledge of the fortification processes.

6 Bangladesh has experience of such incidents, for instance in the oil industry (New Age, 2016).

7 We cover the geographical distribution of the firms, but do not target representation by geography.

8 The unit of analysis in this study is the refinery because production data is for the refinery rather than the enterprise group. However, we assume a single refinery is representative of its enterprise group (in terms of firm behaviour).

9 The starting point to determine appropriate sample sizes is the definition of at least one key indicator on which to base the sample size calculations. We can, and often do, bypass this requirement by setting up a proxy indicator, and estimating the size requirement using a worst case scenario. The margin of error (or sampling error) that represents the range in which the true value of the population is expected to lie is also set. We set a 95% confidence level.

10 60–70% target response rates are typical of firm-level surveys.

11 Respondents could also reply ‘I don’t know’, which was treated as a wrong answer to the question.

12 Note that these five questions together capture firms’ engagement with food fortification – we argue that using any one of these questions, or only a sub-set, would create a potential mis-measurement issue. Hence, we make use of the composite score throughout the paper.

13 In bootstrapping, statistical inference relies on randomly sampling, with replacement, from an original dataset. It can help examine robustness with small sample sizes (Steyerberg et al., 2001).

14 All other things being equal - capturing the effect one economic variable has on another, provided all other variables remain the same.

15 We examine the correlation between our process measure and product outcomes at an aggregate level. We compared the proportion of firms that are identified as complying based on our scores with a proxy for the level of fortification in Bangladesh. First, with respect to oil, the percentage of both bulk and packaged oil that is fortified (either below or above the standard) is 59% (GAIN and icddr’b, 2017). Second, for salt, a more telling statistic is given by the proportion of households that consume fortified product compared to the total population with access to salt, which is 69% (Icddr’b, 2015). Now, according to our scores, the proportion of firms that adequately fortify food in our sample is 66% for oil and 77% for salt respectively. These values are highly correlated with the aforementioned proxies. Further, our scores are also much more accurate than firms’ reported level of compliance in the survey (91% for oil and 90% for salt respectively) that demonstrates that the inaccuracies in the above relationship are compensated for by ease of implementation of our methodology.

16 The relatively small sample (35 observations) may lead to a lack of precision of the estimates. However, we conducted a Wald test for the joint significance of the three primary variables in Model IV and we find these are statistically significant at the 5% level (p=0.025), and thus should be included in the specification.

17 The absence of a statistically significant effect might be partially due to the relative low value of the penalty set out in the current law for salt mills that do not meet fortification requirements (Bangladeshi Taka (BDT) 50,000 or approximately 590 USD).

18 Set at below the third quintile of the variable – splits the sample between those that have full trust in the institutional regime (45 observations) and those that perceive the incentives and penalties already in place as not entirely effective (57 observations).

19 As the dependent variable now is a fractional one, we estimate our models using GLM with the binomial as the distributional family and a logit as the link function, as suggested by Papke and Wooldridge (1996).

20 The dummyComply based on the CBS 1 and the one based on the CI are equivalent.

21 Standard for salt: 20–50 ppm (See for details: https://fortificationdata.org/). Standard for oil: 15–30 Retinol equivalents (RE) ppm (Jungjohann et al., 2021).

22 Note that this proxy is meant to capture not just the level of micronutrient used, but if the firm is correctly fortifying or not and it does not rely on information used to build our compliance score.

23 Health and Safety Executive, UK. Wright, M., S., (1998).
